# Study on Lung Injury Caused by Fine Particulate Matter and Intervention Effect of *Rhodiola wallichiana*

**DOI:** 10.1155/2022/3693231

**Published:** 2022-04-06

**Authors:** Lei Cao, Hua Lin, Qi Li, Shuzhi Han, Hang Yin, Ning Zhang, Yanfeng Gao, Ye Chen, Fen Ping

**Affiliations:** ^1^The Third Department of Geriatrics, Hebei General Hospital, Shijiazhuang City, Hebei 050000, China; ^2^Medical Affairs Office, Hebei General Hospital, Shijiazhuang City, Hebei 050000, China

## Abstract

**Objective:**

The objective of this study was to observe the protective effect of *Rhodiola wallichiana* drops in a rat model of fine particulate matter (PM2.5) lung injury.

**Methods:**

Forty male Wistar rats were randomly divided into blank control (NC), normal saline (NS), PM2.5-infected (PM), and *Rhodiola wallichiana* (RW) groups. Rats in the NC group were not provided any interventions, whereas those in the NS and PM groups were administered normal saline and PM2.5 suspension by trachea drip once a week for four weeks. Rats in the RW group were intraperitoneally administered *Rhodiola wallichiana* for 14 days and then administered PM2.5 suspension by trachea drip 7 days after drug delivery. The levels of inflammatory factors such as interleukin-6, interleukin-1*β*, and tumor necrosis factor-alpha and oxidative stress biomarkers such as 8-hydroxy-2′-deoxyguanosine, 4-hydroxynonenal, and protein carbonyl content were determined in the serum and bronchoalveolar lavage fluid by ELISA. The level of 4-hydroxynonenal in the lung was also determined using Western blotting and immunohistochemical staining.

**Results:**

Levels of inflammatory factors and oxidative stress biomarkers were all increased in the PM group but decreased in the RW group. Western blotting revealed increased 4-hydroxynonenal levels in the PM group but decreased levels in the RW group. Immunohistochemical staining also provided similar results.

**Conclusion:**

*Rhodiola wallichiana* could protect rats from inflammation and oxidative stress injury caused by PM2.5.

## 1. Introduction

Environmental pollution is the largest cause of premature death and disability in the world today [1]. Particulate matter with an aerodynamic diameter of 2.5 microns or less (PM2.5) is the main reason for polluting the air. Analysis of the Global Burden of Disease revealed that PM2.5 was the fifth major risk factor for mortality. Exposure to PM2.5 was the reason for 4.2 million deaths and 103 million incapacitation cases, accounting for 7.6% of the global death toll and 4.2% of the global disability-adjusted life years [2].

PM2.5 affects the respiratory system and leads to systemic damage. Pulmonary inflammation and oxidative stress are the earliest manifestations after PM2.5 invasion, which may then impair blood coagulation cascades and cardiovascular systems and alter the systemic inflammatory response and oxidative stress levels [3]. Exposure to PM2.5 increases the risk of respiratory diseases, including asthma, bronchitis, chronic obstructive pulmonary disease, lung cancer, and pulmonary fibrosis [4–10]. Oxidative stress and inflammatory injury mechanisms contribute to multiple injury pathways of PM2.5 [11–13]. Drugs targeting these mechanisms may protect against the cascading harmful effects of PM2.5 air pollution in humans [14, 15].

*Rhodiola wallichiana* has long been used in traditional Tibetan medicine. It is a perennial plant of the family Crassulaceae that grows on snowy plateaus. Phytochemical and pharmacological studies of this plant have revealed that *Rhodiola crenulata* has obvious cardioprotective, antihypoxic, antifatigue, anti-low temperature, antitumor, antioxidant, radioprotective, neuromodulatory, immunomodulatory, anti-aging, antipulmonary fibrosis, and antirenal fibrosis effects [16]. However, the effect of *Rhodiola wallichiana* in lung injury caused by PM2.5 has not been studied.

Therefore, in this study, a rat model of PM2.5-induced lung injury was established and the protective effect of *Rhodiola wallichiana* was determined by measuring the levels of various inflammatory and oxidative stress biomarkers in the serum and bronchoalveolar lavage fluid (BALF) and by analyzing histopathological changes in the lung tissue of rats.

## 2. Materials and Methods

### 2.1. Instruments and Reagents

Low-temperature (−80°C) refrigerator (Thermo, USA), automatic enzyme standard instrument (Thermo), desktop high-speed low-temperature centrifuge (Thermo), optical microscope (Olympus, Japan), photomicrographic device (Olympus), electrothermal constant temperature box (Shanghai FUMA Experimental Equipment Company), DYY III 40B transfer membrane tank, DYCZ-24D vertical electrophoresis, DYY-7C electrophoresis apparatus, WD-9405A rockers (Beijing Liuyi Instrument Factory), pentobarbital sodium (Beijing Kulaibo Technology Co., Ltd.), rat interleukin-6 (IL-6) ELISA kit (Wuhan Liuhe Biological Co., Ltd.), rat interleukin-1*β* (IL-1*β*) ELISA kit (Wuhan Liuhe Biological Co., Ltd.), rat tumor necrosis factor-alpha (TNF-*α*) ELISA kit (Wuhan Liuhe Biological Co., Ltd.), rat 4-hydroxynonenal (4-HNE) ELISA kit (Shanghai Lanji Biotechnology Co., Ltd.), rat 8-hydroxy-2′-deoxyguanosine(8-OHdG) ELISA kit (Shanghai Lanji Biotechnology Co., Ltd.), rat protein carbonyl content (PCC) assay kit (Nanjing Institute of Biological Engineering), rabbit anti-*β*-actin (Sangon D110001), rabbit anti-4-HNE (Abcam Company, ab46545), *Rhodiola wallichiana* injection (Tonghua Yusheng Pharmaceutical Co., Ltd.), SP immunohistochemical kit (Beijing Zhongshan Reagent Co., Ltd.), and diaminobenzidine staining kit (Fuzhou Maixin Biotechnology Co., Ltd.) were used in the experiments.

### 2.2. PM2.5 Suspension

PM2.5 was provided by the Environmental Monitoring Center of Hebei Province. After high-pressure sterilization, the PM2.5 suspension was prepared at a concentration of 7.5 mg/ml in sterile water for injection; ultrasound oscillation was used for homogenization.

### 2.3. Experimental Groups and Drug Treatment

Forty male Wistar rats (190–220 g; Hebei Medical University Laboratory Animal Center) were procured. They were not used in the experiment until they weighed 250 g by conventional breeding (Experimental site: Laboratory of Scientific Research Center, Hebei General Hospital). All animals were treated and cared for in accordance with the laboratory animal guidelines of Hebei Medical University. All animal experiments complied with the guidelines of the Ethics Committee of Hebei General Hospital, China. By using the method of random number table, the rats were divided into blank control (NC), normal saline (NS), PM2.5-infected (PM), and *Rhodiola wallichiana* (RW) groups. Rats in the NC group were not provided any intervention, whereas those in the NS and PM groups were, respectively, administered normal saline (1 ml/kg) and PM2.5 suspension (1 ml/kg; diluted with sterile water for injection from 7.5 mg/ml stock suspension) by trachea drip once a week for 4 weeks. Rats in the RW group were intraperitoneally administered *Rhodiola wallichiana* (4 ml/kg) once daily for 14 days. A week after the drug delivery, they were administered PM2.5 suspension by trachea drip once a week for four weeks. During the experiment, the general condition of the rats was observed. All rats were killed 24 hours after the last trachea drip.

### 2.4. Evaluation of the PM2.5-Induced Lung Injury Model

After weighing each rat, it was anesthetized with intraperitoneal pentobarbital sodium (40 mg/kg) and laid on its back on an operating table with a 30–40 slope. The teeth and limbs of the rat were restrained in position, and a lamp was used to vertically illuminate the neck. The tongue was allowed to stick out of the mouth and held in position. Under the illumination of the glottis, a No. 12 lavage needle was inserted into the trachea and a certain volume of PM2.5 suspension was dripped. The sound of moist rales and the appearance of bubbles emerging from the windpipe indicated successful tracheal dropping. The lavage needle was then pulled out, and the rat was placed away from the operating table after 10 minutes. It was allowed to recover from the anesthesia by maintaining it in the supine position.

### 2.5. Specimen Collection

All rats were killed 24 hours after the last trachea drip (rats in the NC group were killed 4 weeks after the start of the experiment) by anesthetizing them with intraperitoneal pentobarbital sodium (50 mg/kg) and then subjected to exsanguination through abdominal aorta bleeding. Blood samples were collected from the abdominal aorta and centrifuged at 3000 rpm for 10 minutes. The separated serum samples were stored in a low-temperature refrigerator at −80°C until analysis. The trachea and the main bronchus were separated, and the right main bronchus was clamped with hemostatic forceps. Through a small oblique cut on the trachea, a No. 16 gavage-feeding needle was inserted and fixed with a suture. BALF was aspirated using 5 ml phosphate buffer saline (PBS) thrice. The obtained BALF was centrifuged at 1500 rpm for 10 minutes at 4°C, and the supernatant was stored in a low-temperature refrigerator at −80°C until analysis. Lung tissue of the right lower lobe of the rats that were not subjected to BALF aspiration was fixed with formaldehyde solution, embedded in conventional paraffin, sectioned, and stained with hematoxylin-eosin (HE) or subjected to immunohistochemical staining. Pathological changes were observed under an optical microscope. The rest of the right lung was stored in liquid nitrogen and transferred to the −80°C refrigerator for cryopreservation and preparation of lung tissue homogenate.

### 2.6. Biochemical Analysis

#### 2.6.1. Western Blot Analysis of 4-HNE Levels in Lung Tissue

The lung tissue was minced and ground with liquid nitrogen, mixed with cell lysis solution, and homogenized. The protein concentration in the supernatant obtained after centrifugation was measured using the BCA Protein Quantification Kit. Samples of the lung tissue supernatant containing 30 *μ*g protein were loaded in each well of the Western blotting apparatus. The sample buffer was added, and the mixture was heated to 95°C for 5 minutes to denature the proteins and then cooled rapidly on ice. The SDS-PAGE reaction was stopped when the bromophenol blue indicator moved to the bottom of the separation gel. Separated proteins were then transferred to a PVDF membrane under a constant current of 190 mA; the transfer time was selected based on the molecular weight of the target protein. After blocking the membrane with nonfat milk and washing with TTBS, the membrane was incubated overnight with the primary antibodies against 4-HNE (1 : 1000) and *β*-actin (1 : 1000). After washing, the membrane was incubated with anti-rat (1 : 2000) and anti-rabbit (1 : 5000) secondary antibodies for 1.5 hours. After washing the membrane, the protein bands were visualized by the electrochemiluminescence method. Densitometric analysis was performed using the Gel-Pro analyzer software.

#### 2.6.2. Immunohistochemical Analysis of 4-HNE

For immunohistochemical staining, all samples were sectioned into 5 *μ*m slices. Briefly, after blocking, the sections were incubated with antibody against 4-HNE (1 : 100) overnight at 4°C. Visualization was achieved with peroxidase‐labeled streptavidin‐biotin and diaminobenzidine staining (1 hour at room temperature). Stain-positive cells were observed in high‐power fields by using a light microscope (×200 objective).

#### 2.6.3. Quantification of Biomarkers of Inflammation and Oxidative Stress

IL-6, IL-1*β*, TNF-*α*, 8-OHdG, and 4-HNE were quantified in the serum and BALF of rats by using their respective ELISA kits. PCC was quantified by using UV-Vis spectrophotometry according to the test kit instructions.

### 2.7. Statistical Analysis

All data were tested for normality by the Shapiro–Wilk test, and all values are presented as the mean ± standard deviation (mean ± SD). All statistical analyses were conducted using SPSS 21.0 Statistics Software. Multiple groups were compared by using one-way ANOVA followed by Student–Newman–Keuls post hoc tests for different pairwise comparisons. A difference was considered statistically significant if *P* < 0.05.

## 3. Results

### 3.1. General Condition of Animals

The physical state of rats in the NC and NS groups was good. They had a lustrous coat, showed a constantly increasing bodyweight, responded quickly to external stimulus, and had a normal breathing pattern and diet. Rats in the PM group showed low mobility and did not effectively resist handling by the researchers. They had a yellow, dry coat and exhibited slow weight gain. Shortness of breath with increased nasal and oral secretions after tracheal drip was noticed. The condition of rats in the RW group was similar to the condition of rats in the PM group, but the negative effects of PM2.5 were less severe in this group.

### 3.2. Lung Histopathology

**Figure 1:**
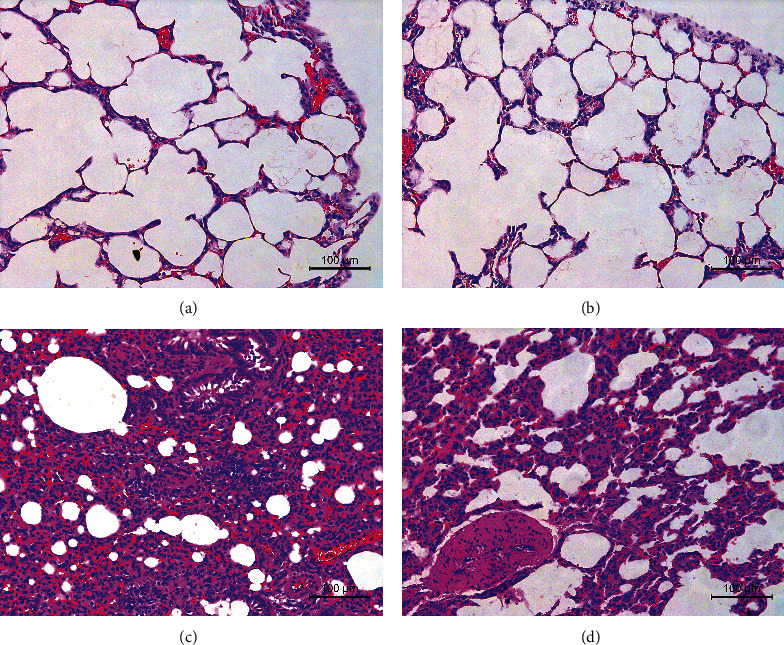
(a–d) Pathological sections of rat lung tissues in each group (HE staining, 200×): NC group (a), NS group (b), PM group (c), and RW group (d).

As shown in Figures 1(a)–1(d), alveolar cells were uniformly distributed and had a complete structure in the NC and NS groups. There was no obvious inflammatory cell infiltration, no expansion of congestion between alveolar capillaries, and no interstitial edema. In the PM group, the alveolar septum was markedly thickened. Many disrupted alveolar networks and severe interstitial edema were visible. Severe inflammatory cell infiltration within the bronchial wall, vascular cavity, and small airway, and hemorrhage in the alveolar cavity were observed. The pathologic changes in the lung tissue of rats in the RW group were less severe than those in the PM group.

### 3.3. Biochemical Changes in the Serum

**Figure 2:**
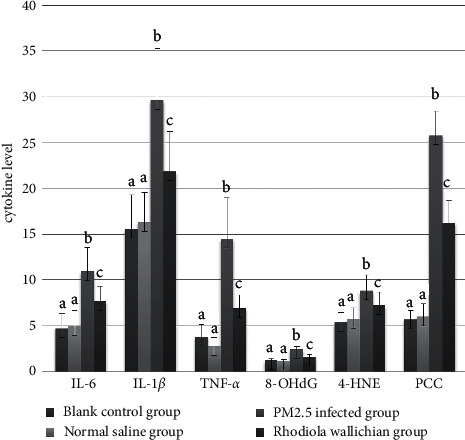
Comparisons of IL-6, IL-1*β*, TNF-*α*, 8-OHdG, 4-HNE, and PCC in the rat serum of each group; cytokine level is described as mean ± SD (*n* = 10); the same letters indicate nonstatistical differences, *P* > 0.05; different letters indicate statistical differences, *P* < 0.05.

Serum levels of IL-6, IL-1*β*, TNF-*α*, 8-OHdG, 4-HNE, and PCC differed significantly among the NC, NS, PM, and RW groups (*P* < 0.001; Figure 2, Table 1). Between-groups analysis confirmed that the levels were higher in the PM and RW groups than in the NC and NS groups (*P* < 0.05), whereas they were lower in the RW group than in the PM group (*P* < 0.05). The NC and NS groups did not differ regarding these levels (*P* > 0.05).

### 3.4. Biochemical Changes in BALF

**Figure 3:**
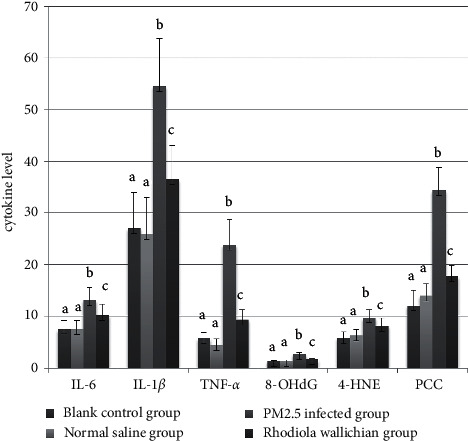
Comparisons of IL-6, IL-1*β*, TNF-*α*, 8-OHdG, 4-HNE, and PCC in the BALF of rats in each group; cytokine level is described as mean ± SD (*n* = 10). Same letters indicate nonstatistical differences, *P* > 0.05; different letters indicate statistical differences, *P* < 0.05.

Levels of IL-6, IL-1*β*, TNF-*α*, 8-OHdG, 4-HNE, and PCC in the BALF differed significantly among the NC, NS, PM, and RW groups (*P* < 0.001; Figure 3, Table 2). Between-groups analysis confirmed that the levels were higher in the PM and RW groups than in the NC and NS groups (*P* < 0.05), whereas they were lower in the RW group than in the PM group (*P* < 0.05). The NC and NS groups did not differ regarding these levels (*P* > 0.05).

### 3.5. Levels of 4-HNE in the Rat Lung Tissue

#### 3.5.1. Western Blotting Results

**Figure 4:**
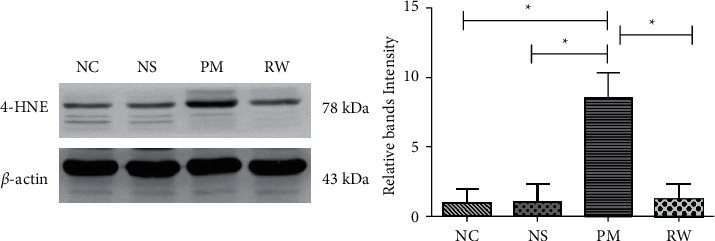
Western blotting showed that 4-HNE expression in the PM group increased and decreased after *Rhodiola wallichiana* intervention (*P* < 0.05), and the difference between the NC group and the NS group was not significant (*P* > 0.05). “^*∗*^” in the figure indicates a statistical difference, *P* < 0.05.

The protein expression of 4-HNE was significantly higher in the PM group than in the NC and NS groups (*P* < 0.05; Figure 4), whereas it was lower in the RW group than in the PM group (*P* < 0.05). This indicated that *Rhodiola wallichiana* decreases the expression of 4-HNE. The NC and NS groups did not significantly differ regarding the level of 4-HNE (*P* > 0.05).

#### 3.5.2. Immunohistochemical Staining Results

**Figure 5:**
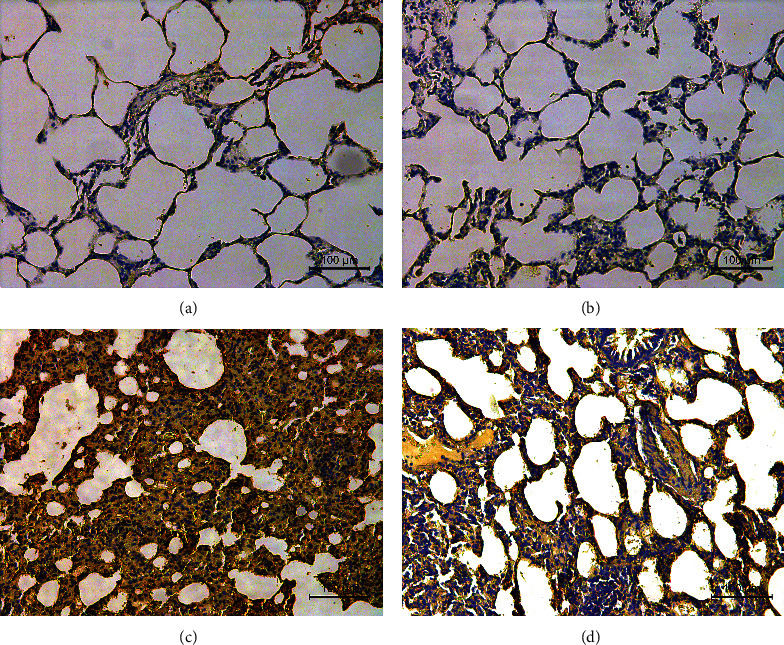
(a–d) 4-HNE expression levels in each group of rats with immunohistochemical staining (200*x*): NC group (a), NS group (b), PM group (c), and RW group (d). 4-HNE in the PM group was obviously higher than that in the NC and NS groups. 4-HNE expression of the RW group was lower than that of the PM group, which suggested that 4-HNE expression decreased after administration of *Rhodiola wallichiana*.

**Figure 6:**
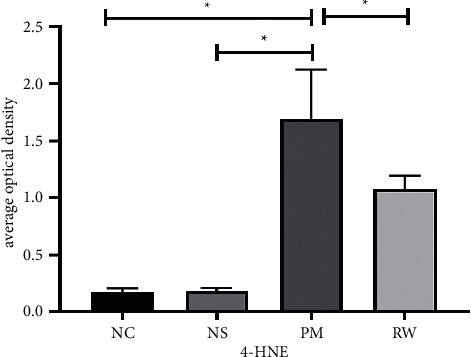
Expression of 4-HNE in each group of rats by immunohistochemical staining analysis (average optical density). 4-HNE in the PM group was obviously higher than that in the NC and NS groups. 4-HNE expression of the RW group was lower than that of the PM group. “^*∗*^” in the figure indicates a statistical difference, *P* < 0.05.

The reaction was considered positive based on the presence of specific tan or brown granules in the cytoplasm (Figures 5(a)–5(d), Table 3, and Figure 6). The optical density was determined on five randomly selected fields for each section, and the average optical density was used to indirectly estimate the relative 4-HNE expression. Immunohistochemical staining results were consistent with the Western blotting results. The levels were higher in the PM and RW groups than in the NC and NS groups (*P* < 0.05), whereas they were lower in the RW group than in the PM group (*P* < 0.05), suggesting that 4-HNE expression decreased after administration of *Rhodiola wallichiana*.

## 4. Discussion

Air pollution has become a major global environmental and public health risk, which increases disease burden, morbidity, and mortality and reduces life expectancy. Fine particulate matter (PM2.5) poses the greatest health risk and is implicated in various acute and chronic diseases of the lungs. According to a systematic review and meta-analysis study on the relation of PM2.5 with all-cause mortality and hospitalization, for every 10 *μ*g/m^3^ increase in PM2.5, the risk of death increases by 1.04%, and death due to respiratory diseases death is more common than that due to other diseases. In addition, air pollution levels significantly differ in regions worldwide, and only 0.4% of China's population lives in areas meeting WHO air quality standards [17]. In 2017, a study from China expounded the relation between PM2.5 concentrations and cause of death due to diseases of the respiratory tract, and the results confirmed that for one-day lag and for every 10 *μ*g/m^3^ increase in PM2.5 concentration, respiratory death increased by 0.30%, whereas for a longer-time lag, the corresponding increase in death due to respiratory diseases was 0.69% [18].

The toxicity of PM2.5 is mainly related to its complex chemical composition [19]. Heavy metals including Pb, Mn, Cd, Cr, and Ni have been detected in PM2.5 along with polycyclic aromatic hydrocarbons, which are carcinogenic components of PM2.5. Metabolomics analysis has demonstrated that PM2.5 exposure not only significantly alters the lung microbiome composition but also perturbs the levels of many metabolites involved in diverse metabolic pathways, thus damaging multiple organs [20, 21].

Immune and inflammatory responses, oxidative stress, and DNA damage are potential mechanisms contributing to the adverse health effects of PM 2.5 [22–25]. The lung is the direct target organ for inhalational damage caused by PM2.5. PM2.5 can damage the entire respiratory system, generate reactive oxygen species, and trigger the release of inflammatory mediators that can induce DNA adduct formation, ultimately leading to obstructive or restrictive respiratory diseases [26]. PM2.5 mainly induces lung injury through increased inflammatory response and oxidative stress damage including DNA damage, lipid peroxidation, and cell death [27–30]. When human bronchial epithelial cells (BEAS-2B) were treated with PM2.5, the gene expression of inflammatory factors such as TNF-*α*, IL-1*β*, IL-6, and IL-8 increased proportionally to the exposure duration and concentration. Animal experiments have demonstrated that PM2.5 can cause tissue congestion and inflammatory cell infiltration in the lungs, elevated white blood cells and macrophages in the BALF, and upregulation of proinflammatory mediators such as TNF-*α*, IL-6, and IL-1*β* in the lungs and serum [31, 32]. In 2019, it was shown for the first time that prolonged exposure to PM2.5 alone can cause chronic obstructive pulmonary disease in mice, and long-term PM2.5 exposure increases the expression of proinflammatory cytokines (IFN-*γ*, TNF-*α*, IL-17A, IL-6, and IL-8) associated with the condition in the mouse serum and BALF. Long-term exposure to PM2.5 can lead to emphysema, decreased lung function, pulmonary inflammation, and systemic inflammation [33]. Mice exposed to PM2.5 for 3 months had increased levels of DNA lesions related to oxidative stress in the lungs, liver, and kidney and had decreased global DNA methylation levels in the lung and liver DNA [25]. PM2.5 exposure can cause changes in superoxide dismutase, glutathione, malondialdehyde, and total antioxidant capacity, causing oxidative stress damage to the lung [34, 35]. In 2020, Yangde et al. found that RAB6 deficiency attenuates PM2.5-induced lung injury and fibrosis in mice by inhibiting alveolar epithelial cell death and oxidative stress in the lung tissue of PM2.5-exposed mice [36].

Pretreatment with vitamin E and omega-3 polyunsaturated fatty acids in rats exposed to PM2.5 improves antioxidant activity and reduces the production of inflammatory cytokines [37]. Resveratrol can also reduce the degree of lung inflammation and fibrosis in rats [38]. In a recent clinical study in China, healthy college students were asked to consume 2.5 g of fish oil daily during their exposure to PM2.5; the study found that fish oil protected against inflammation, blood clotting, endothelial dysfunction, oxidative stress, and neural endocrine disorder, verifying the cardiovascular health benefits of fish oil [39]. Therefore, we speculated that pretreatment of drugs with anti-inflammatory and antioxidant mechanisms can reduce the organ damage caused by PM2.5.

*Rhodiola wallichiana* belonging to Rhodiola rosea genus and Crassulaceae family mainly contains salidroside, tyrosol, Rhodiola Finn, rozarin, polysaccharide, flavonoids, 18 amino acids, and 21 trace elements. According to traditional Chinese medicine, it promotes blood circulation to remove blood stasis, clears veins, and relieves pain, supporting healthy energy, clearing away heat and toxic materials, and removing heat from the lung to relieve cough. It is widely used in the clinical treatment of cardiovascular and cerebrovascular diseases. Rhodiola wallichiana injection in patients with acute coronary syndrome improves indexes of oxidative stress and inflammation. Meanwhile, Rhodiola rosea significantly reduces levels of markers of oxidative stress in the blood and normalizes peak strain rate in breast cancer patients [40]. *Rhodiola sp.* can reduce TNF-*α*, transforming growth factor beta 1, and IL-6 levels in the BALF in a concentration-dependent manner and inhibit inflammatory injury caused by cerebral ischemia in rats, slowing the progression of brain edema [41]. By inhibiting oxidative stress caused by free radicals, *Rhodiola sp.* protects against UVB-induced premature senescence of human dermal fibroblasts [42]. Toxicological studies have established the safety of *Rhodiola rosea*. Because the plant protects against organ damage, we hypothesized that *Rhodiola rosea* can attenuate inflammatory and oxidative stress damage due to PM2.5 exposure. A literature review revealed no relevant studies on this topic. We used the trachea drip method to establish a rat model of PM2.5-induced lung injury to observe the protective effect of *Rhodiola wallichiana*.

Several epidemiological studies have confirmed a concentration-response relationship between airborne PM2.5 and its deleterious effects on the respiratory system. A recent study on mice found that the severity of lung injury induced by environmental PM exposure is related to its cumulative dose. Acute exposure to low doses of fine PM by nasal drip induces pulmonary inflammation and oxidative stress in a dose-dependent manner [43]. Several animal studies have adopted the method of tracheal PM2.5 drip, and the dosing schedules reported are 0.2–2.7 mg/kg every 3 days for 2 months with a maximum cumulative dose of 54 mg/kg, 1.8–16.2 mg/kg every 3 days for 1 month, and 10–40 mg/kg once a week for up to 12 weeks. Based on the daily respiratory volume of rats (about 0.288 m^3^) and the average concentration of PM2.5 in our city (as high as 1000 *μ*g/m^3^ in winter, with an average of 600 *μ*g/m^3^), the total weekly exposure dose of PM2.5 in rats was calculated. Considering the bodyweight of an adult rat as 250 g, the PM2.5 exposure concentration was approximately 5 mg/kg once every 7 days. Therefore, we administered PM2.5 at three dose levels of 5, 7.5, and 10 mg/kg for the preliminary experiment and found that the intermediate dose could produce significant toxic effects without any significant behavioral abnormalities, changes in bodyweight, and death. With this dosing regimen, the lung tissue of rats showed severe lung injury compared with the NC group [44–47].

Trachea drip of PM2.5 resulted in abnormal changes in weight, color, activities, and mental state of rats compared with the NC group. These changes were less severe in the RW group than in the PM group. HE staining revealed thickened and fused alveolar intervals and many fractured alveolar intervals in the PM group. Interstitial edema, capillary expansion, and massive infiltration of inflammatory cells such as mononuclear cells, lymphocytes, and neutrophils in the bronchial wall, vascular cavity, and small airway were also noted along with bleeding in alveolar cavities. Alveolar destruction and inflammatory cell infiltration were less severe in the RW group than in the PM group. Moreover, levels of IL-6, IL-1*β*, and TNF-*α* in the serum and BALF were significantly higher in the PM group than in the RW group, which supports the anti-inflammatory effect of *Rhodiola wallichiana*.

Oxidative stress is an important mechanism of lung injury caused by PM2.5. PCC is the most commonly used biomarker to evaluate protein oxidative damage, whereas 8-OHdG is a specific biomarker of DNA oxidative damage caused by various endogenous and exogenous factors. 4-HNE is the product of oxidative damage and indicates lipid peroxidation. All three abovementioned biomarkers indicate the extent of oxidative stress. In another study, the levels of 8-OHdG and 4-HNE in the BALF of rats with the chronic obstructive pulmonary disease were increased [48]. Similarly, we found increased levels of 8-OHdG, 4-HNE, and PCC in the serum and BALF of rats in the PM group, which indicates that fine PM impairs the antioxidant system and induces lipid peroxidation and oxidative DNA damage. These levels were significantly lower in the RW group than in the PM group. Western blotting and immunohistochemical analysis revealed increased 4-HNE levels in the lung tissue of rats in the PM group compared to those in the RW group, which supports the antioxidant effect of *Rhodiola wallichiana*.

In conclusion, the results of this study show that *Rhodiola wallichiana* can alleviate PM2.5-induced lung injury to a certain degree in terms of pathological morphological changes in the lung tissue and levels of inflammatory and oxidative stress biomarkers in the serum and BALF. Therefore, such drugs can be studied for their clinical potential to prevent and control lung injury caused by PM2.5 in polluted air.

## 5. Conclusion

PM2.5 exposure can lead to inflammatory and oxidative stress damage in the serum and lung tissues of rats. Animal experiments have shown that early *Rhodiola wallichiana* intervention has a certain protective effect on this injury, which may be due to the downregulation of the levels of inflammatory and oxidative stress factors caused by PM2.5 exposure. This study provides a theoretical basis for developing effective clinical therapeutic drugs against oxidative stress injury caused by PM2.5.

## Figures and Tables

**Table 1:** Comparison of cytokine level in the serum of four groups of rats (*n* = 10).

Group	IL-6 (pg/ml)	IL-1*β* (pg/ml)	TNF-*α* (pg/ml)	8-OHdG (ng/ml)	4-HNE (ug/ml)	PCC (nmol/mg.prot)
Blank control group	4.725 ± 1.628^a^	15.605 ± 3.673^a^	3.797 ± 1.337^a^	1.238 ± 0.130^a^	5.359 ± 1.031^a^	5.679 ± 1.012^a^
Normal saline group	4.980 ± 1.690^a^	16.282 ± 3.29^a^	2.774 ± 0.923^a^	1.190 ± 0.127^a^	5.767 ± 1.180^a^	5.990 ± 1.402^a^
PM2.5-infected group	10.978 ± 2.572^b^	29.650 ± 5.611^b^	14.513 ± 4.451^b^	2.457 ± 0.296^b^	8.845 ± 1.695^b^	25.782 ± 2.644^b^
Rhodiola wallichiana group	7.690 ± 1.660^c^	21.858 ± 4.355^c^	6.920 ± 1.432^c^	1.633 ± 0.223^c^	7.242 ± 1.465^c^	16.253 ± 2.486^c^
*F*	22.875	22.619	46.014	80.887	13.420	227.716
*P*	＜0.001	＜0.001	＜0.001	＜0.001	＜0.001	＜0.001

The same letters indicate nonstatistical differences, *P* > 0.05; different letters indicate statistical differences, *P* < 0.05.

**Table 2:** Comparison of cytokine levels in BALF of the four groups of rats (*n* = 10).

Group	IL-6 (pg/ml)	IL-1*β* (pg/ml)	TNF-*α* (pg/ml)	8-OHdG (ng/ml)	4-HNE (ug/ml)	PCC (nmol/mg.prot)
Blank control group	7.547 ± 1.59^a^	26.981 ± 7.051^a^	5.79 ± 1.01^a^	1.299 ± 0.118^a^	5.742 ± 1.169^a^	12.084 ± 3.034^a^
Normal saline group	7.442 ± 1.777^a^	25.938 ± 7.114^a^	4.42 ± 1.185^a^	1.381 ± 0.23^a^	6.251 ± 1.254^a^	13.974 ± 2.285^a^
PM2.5-infected group	13.167 ± 2.143^b^	54.497 ± 9.317^b^	23.773 ± 4.88^b^	2.657 ± 0.387^b^	9.747 ± 1.555^b^	34.387 ± 4.33^b^
Rhodiola wallichiana group	10.139 ± 2.213^c^	36.497 ± 6.526^c^	9.347 ± 1.953^c^	1.633 ± 0.179^c^	8.057 ± 1.56^c^	17.808 ± 2.095^c^
*F*	17.776	30.468	104.765	62.809	17.090	110.016
*P*	＜0.001	＜0.001	＜0.001	＜0.001	＜0.001	＜0.001

The same letters indicate nonstatistical differences, *P* > 0.05; different letters indicate statistical differences, *P* < 0.05.

**Table 3:** Expression of 4-HNE in each group of rats by immunohistochemical staining analysis (average optical density).

Group	*N*	4-HNE
Blank control group	10	0.175 ± 0.029^a^
Normal saline group	10	0.180 ± 0.023^a^
PM2.5-infected group	10	1.673 ± 0.394^b^
Rhodiola wallichiana group	10	1.069 ± 0.117^c^
*F*		112.9
*P*		＜0.0001

The same letters indicate nonstatistical differences, *P* > 0.05; different letters indicate statistical differences, *P* < 0.05.

## Data Availability

The image data used to support the findings of this study are included in the article.
